# Influence of construction and demolition waste on fitness and community structure of cicada nymphs: New bioindicators of soil pollution

**DOI:** 10.1371/journal.pone.0203744

**Published:** 2018-09-14

**Authors:** Zehai Hou, Yunxiang Liu, Cong Wei

**Affiliations:** Key Laboratory of Plant Protection Resources and Pest Management, Ministry of Education, College of Plant Protection, Northwest A&F University, Yangling, Shaanxi, China; Public Library of Science, UNITED KINGDOM

## Abstract

Construction and demolition (C&D) waste is a novel, widespread environmental stress that negatively affects environment, organisms and ecosystem. Yet effects of cumulative consequences of C&D waste on the fitness and community structure of most underground insects remain unclear. With relatively sessile life underground lasting several years, cicadas can be an important aspect in forest nutrient cycles. Whether cicada nymphs are easily affected by C&D waste, and whether they have evolved any adaptations to cope with the adverse environments merit exploration. Here, we investigated the biodiversity, community structure, population dynamics and morphology of cicada nymphs in both uncontaminated and contaminated habitats by C&D waste in Guanzhong Plain, China since 2011. In total, 1,573 cicada nymphs were collected from 2011 to 2015, including 62 malformed nymphs. The malformed nymphs can be divided into three types: physically damaged individuals (still alive) (3.2%), fungus-infected individuals (dead) (64.5%), and bacterium-infected individuals (dead) (32.3%). The proportion of malformed nymphs increased year by year in the habitats contaminated by C&D waste. In the uncontaminated habitats, although no significant differences of population density among the investigated years were observed, yet there was a distinct increasing trend of population of *Meimuna mongolica*, whereas populations of both *Cryptotympana atrata* and *Platypleura kaempferi* distinctly decreased. This indicates that *M*. *mongolica* is possibly evolving into the most dominant species in the ecological niche when it competes with other sympatric species, but more researches are needed to establish whether there is a shift in the species composition of cicadas. In the habitats contaminated by C&D waste, a higher ratio of malformed individuals and a decline of population of both *M*. *mongolica* and *C*. *atrata* were revealed; *P*. *kaempferi* was not found in the contaminated habitats, indicating a weaker resistance of this species against C&D waste. The negative responses of cicada nymphs to C&D waste have significant implications for the habitat destruction. Cicada nymphs may be suitable bioindicators for underground-habitat-quality monitoring, as merits further research to reveal the association between the magnitude of C&D waste contamination with the fitness and population dynamics of cicada nymphs.

## Introduction

Urbanization has greatly facilitated people’s lives, but it is meanwhile a big challenge to environmental conservation. As the world continues to urbanize, sustainable development challenges will be increasingly concentrated in agricultural and forest landscapes, especially in the lower-middle-income countries where the pace of urbanization is the fastest [1].

In the process of urbanization, a large amount of construction and demolition (C&D) waste is generated without proper management, appropriate recycle and reuse, which occupies arable land and causes severe destruction of habitat. For example, on an annual basis, 1.5 billion tons of construction waste is generated in China, but with only 5% recycled and reused, which causes serious environmental pollution and habitat mosaics [2]. Previous studies have confirmed the negative effect of C&D waste on environment and certain plants, animals and human life [3, 4]. However, studies of influence of C&D waste on insects living a long time underground are extremely few.

Cicadas (Hemiptera: Cicadidae) have an immature life stage below ground, usually lasting several years, which is much longer than that of most other insects [5, 6]. With higher population densities cicadas can be an important aspect in forest nutrient cycles [7]. During their subterranean lives, cicada nymphs burrow through soil and feed exclusively on the xylem sap from roots of their host plants [8]. Due to the close association with their host roots, cicada nymphs are relatively motionless underground [9, 10]. Oviposition-site choice of female cicadas greatly determines the long-term subterranean lives of their offspring [11, 12]. Whether C&D waste affects the fitness of cicada nymphs and whether these insects evolved any adaptations to cope with the adverse environments have never been studied.

Herein, we investigated the population dynamics and morphology of cicada nymphs in both uncontaminated and contaminated habitats by C&D waste in Guanzhong Plain, Shaanxi Province, China. We are aiming to evaluate whether C&D waste alters the fitness and community structure of these cicadas. We focused our efforts on the campus of Northwest A&F University, as its annual infrastructure represents a miniature of rapid urbanization of this area.

## Materials and methods

### Study site

**Fig 1:**
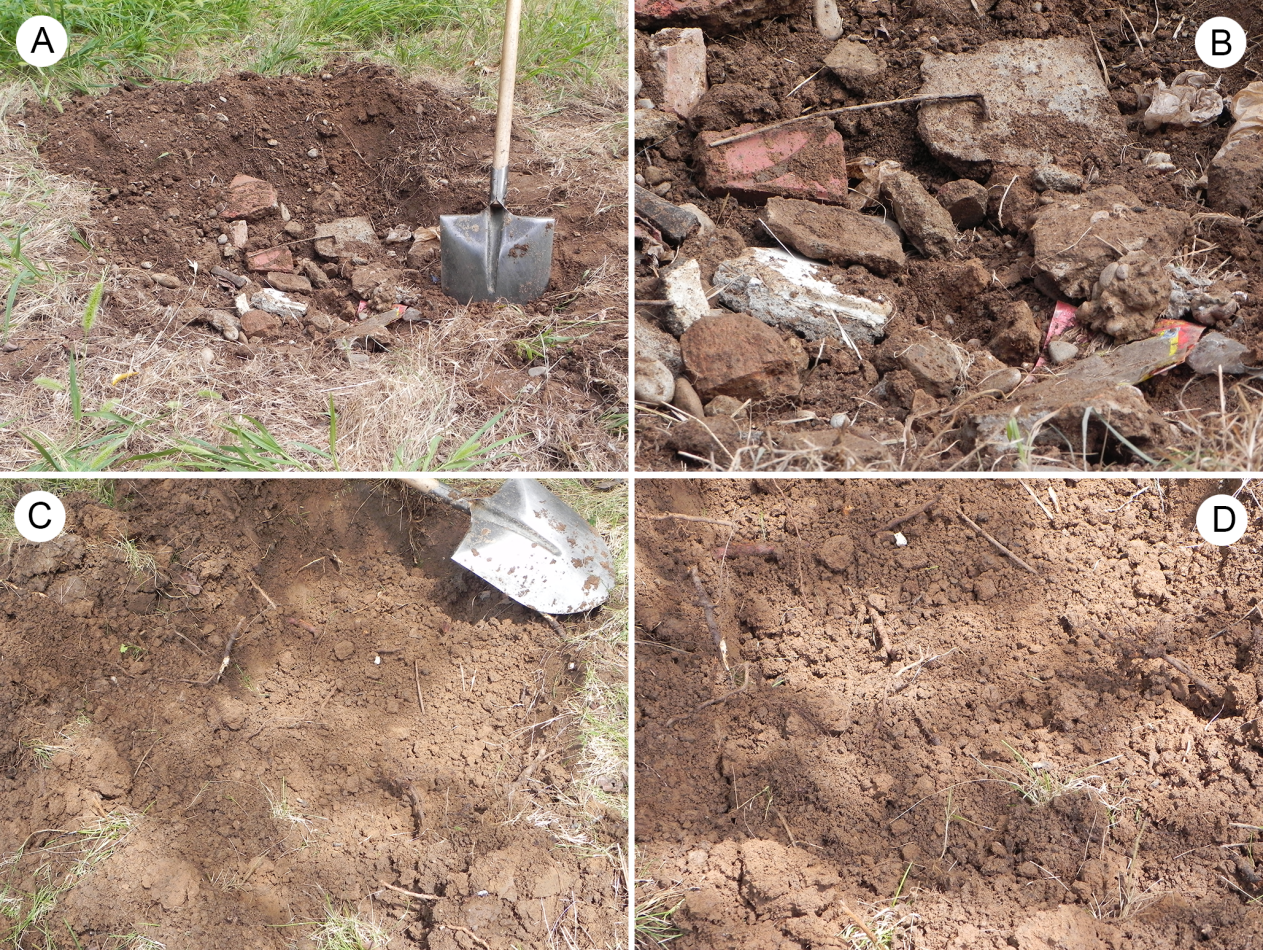
Sample sites. (A) and (B) Contaminated sample sites. (C) and (D) Uncontaminated sample sites.

Field work was carried out on the campus of Northwest A&F University, Yangling, Shaanxi Province, China (34°16′56.24″N, 108°4′27.95″E). Annual infrastructure of the university has produced much waste in building demolition and construction in the past two decades. Components of C&D waste here, produced in the process of construction, renovation and/or demolition of residential or non-residential structures, typically include stone, concrete, lime, gypsum wallboard, broken brick, scrap metals, plastic and broken glass, *etc*. The C&D waste was buried in the soil and mainly distributed in the 20–60 cm soil layer (Fig 1A and 1B). Most of the time C&D waste eventually ended up in landfills, disturbing environmental, economical and social life cycle. Habitats without C&D waste were selected as control sites (Fig 1C and 1D).

The climate of the study sites is semiarid and prone-to-drought. The average annual temperature is 11.0–14.0°C; the average annual rainfall is 650 mm with peaks during July to September; and the average annual evaporation is 800 mm.

### Insects and sampling methods

The biodiversity, community structure, population dynamics and morphology of cicada nymphs were investigated in both uncontaminated and contaminated habitats by C&D waste. A large number of cicada nymphs were excavated and collected from 99 pits under their host plants since the summer of 2011. The woods in our study sites were logged, and the habitats were seriously destroyed in early 2016, resulting in the cease of our investigation.

In detail, 20-pit samplings were carried out in the soil polluted by C&D waste under the host plant *Populus tomentosa*, with 5, 5, 5 and 5 pits in July of 2011, 2013, 2014 and 2015, respectively. Soil sampling was also carried out under host plants *Populus tomentosa*, *Pinus tabuliformis* and *Pyrus xerophila* in uncontaminated habitats. Among which, 23 pits were surveyed under *Populus tomentosa*, with 6, 6, 6 and 5 pits in 2011, 2013, 2014 and 2015, respectively; 37 pits were investigated under *Pinus tabuliformis*, with 12, 12, 8 and 5 pits in 2011, 2013, 2014 and 2015, respectively; 19 pits were investigated under *Pyrus xerophila*, with 8, 6 and 5 pits in 2013, 2014 and 2015, respectively. All pits (1 m × 1 m × 0.7 m) were located randomly under the crown of the host plants [13].

Investigations in 2012 and investigations under the host plant *Pyrus xerophila* in 2011 were interrupted, thus the related, limited data were not included in our analyses.

### Species identification and morphological study

All captured nymphs were transferred to a beaker and anesthetized by chilling at 4°C in a refrigerator. In order to address the identity and to investigate the fitness of nymphs, morphological study was conducted [14]. Observations of the morphological features were carried out using a SMZ168 Stereoscopic Zoom Microscope (Motic, Xianmen, China). Photographs of nymphs were taken with a scientific digital micrography system equipped with an Auto-montage imaging system and a Retiga 2000R digital camera (CCD) (Qimaging, Surrey, BC, Canada).

### Molecular identification

Considering intraspecific morphological variations may occur in related cicada species, especially in the 3^rd^- and 4^th^-instar nymphs [13], the mitochondrial *COI* barcode was employed to further ensure our morphological identification. In total, 64 *COI* sequences (591 bp) from 64 representatives (individuals) were obtained (Table 1). All sequences obtained in this study were submitted to GenBank (accession numbers: KU218583–KU218646).

**Table 1:** Sixty-four representatives of the cicada samples used in the genetic analysis and their identity.

Species	Younger instar nymphs	Final instar nymphs	Adults
*Crypotympana atrata*	12	6	5
*Meimuna mongolica*	10	5	5
*Platypleura kaempfeir*	10	6	5

### Statistical analyses

Population density of nymphs of each species among different years was tested using one-way ANOVA, followed by Student-Newman-Keuls test. Data that did not meet the assumptions required for parametric testing were analyzed using a Kruskal-Wallis ANOVA test and a Dunn-Bonferroni test for post hoc comparisons. Fisher’s exact test was used to detect differences of proportion of malformed nymphs in both uncontaminated soil and contaminated soil among different years.

Data were analyzed using IBM SPSS statistics 20 (IBM, Armonk, NY, USA). In all tests, significance was assessed at α = 0.05, and results were reported as means ± SE.

### Ethics statement

No specific permits were required for this study. This study did not involve endangered or protected species, and *C*. *atrata*, *M*. *mongolica* and *P*. *kaempferi* used in the present study were not included in the “List of Protected Animals in China”.

## Results

### Determination of species

**Fig 2:**
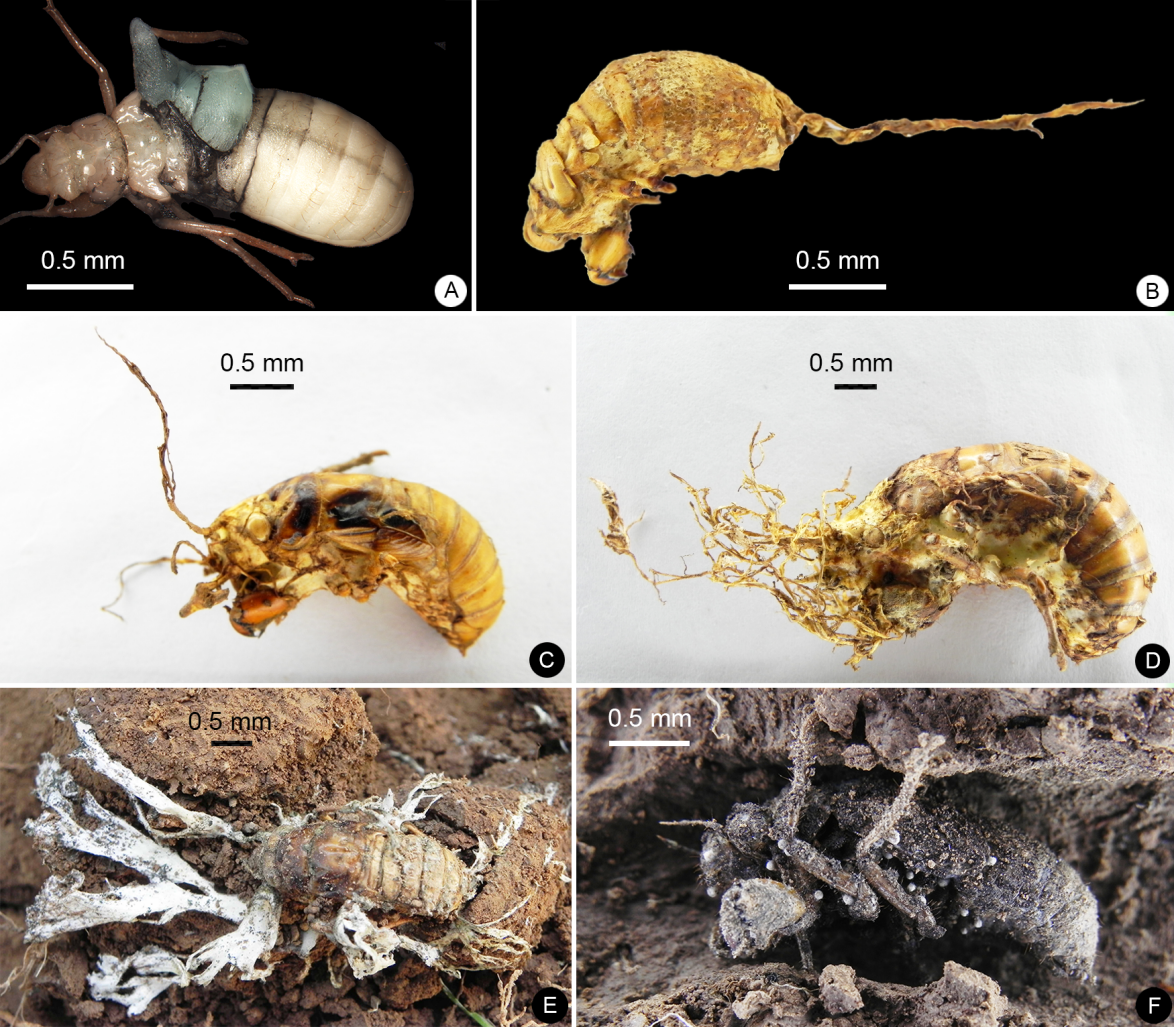
Malformed cicada nymphs. (A) Physically damaged nymphs. (B), (C), (D) and (E) Fungus-infected nymphs. (F) Bacterium-infected nymphs.

In total, 1,573 nymphs were collected from the 99 pits, including 62 malformed nymphs (Fig 2). According to the morphology, they were identified to belong to three species, i.e., *Cryptotympana atrata*, *Meimuna mongolica* and *Platypleura kaempferi*, respectively.

Molecular identification based on the 64 representatives shows that 23, 20 and 21 individuals belong to *C*. *atrata*, *M*. *mongolica* and *P*. *kaempferi*, respectively (Table 1). The *COI* sequences of all individuals within a species determined according to morphology had little divergence. This confirmed the morphological identifications are correct. The intraspecific corrected distances were 0, 0–0.2% and 0 for *C*. *atrata*, *M*. *mongolica* and *P*. *kaempferi*, respectively. None of them reach to interspecific corrected genetic distances according to previous studies, e.g., over 0.2% in the cicada genus *Cicadetta* Kolenati [15], and over 0.5% in the cicada genus *Mogannia* Amyot & Audinet-Serville [16].

### Population dynamics of nymphs in contaminated soil

**Fig 3:**
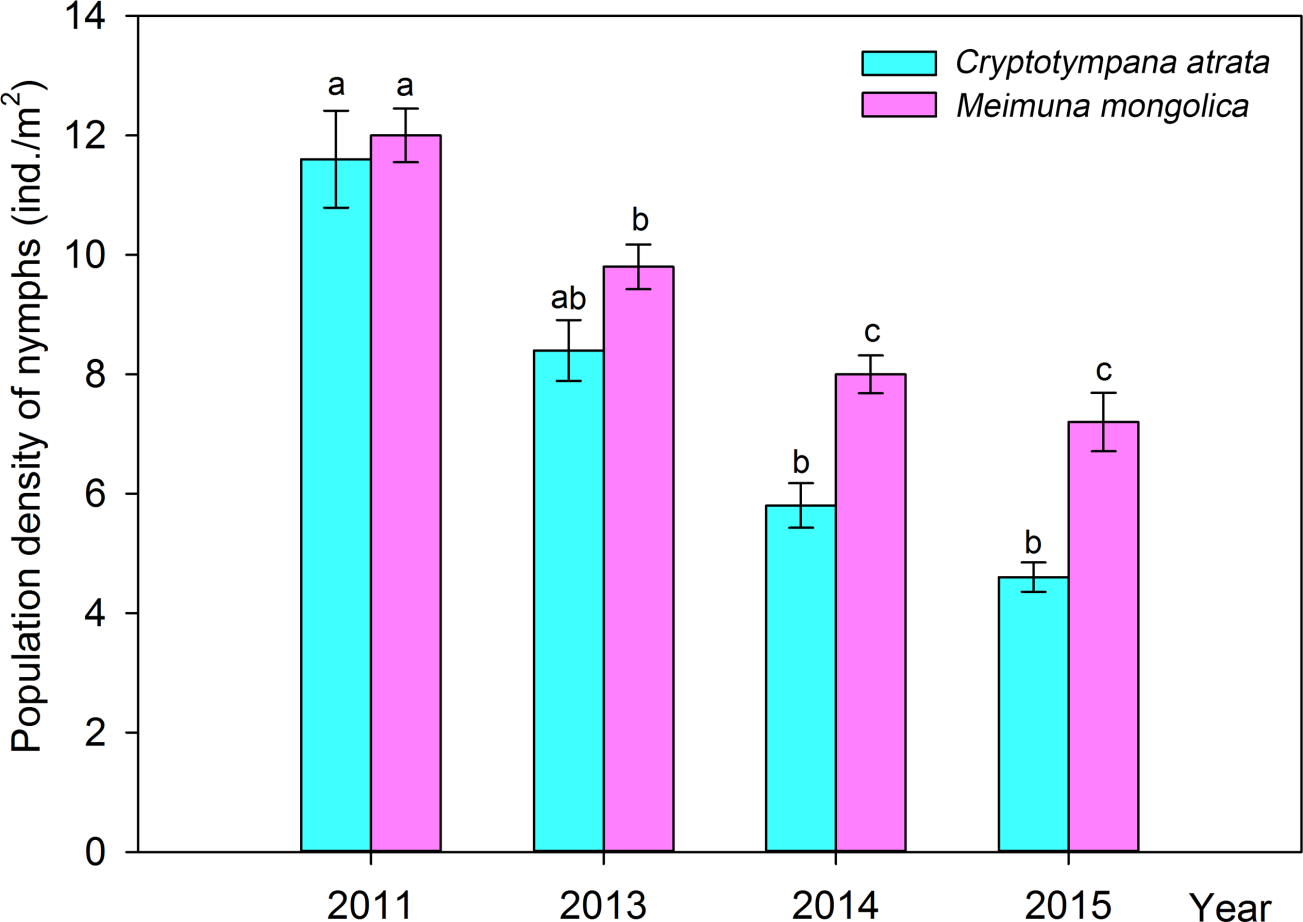
Population dynamics of nymphs of *C*. *atrata* and *M*. *mongolica* feeding on *Populus tomentosa* in habitats contaminated by C&D waste. Different letters represent significant differences at the 0.05-level.

In the soil contaminated by C&D waste in the woods of *Populus tomentosa*, the population of *C*. *atrata* nymphs decreased year by year (Fig 3), with highly significant differences (Kruskal-Wallis test, H = 17.024, P = 0.001). The population of *M*. *mongolica* nymphs also significantly decreased year by year (ANOVA, F = 26.725, P < 0.001) (Fig 3).

### Population dynamics of nymphs in uncontaminated soil

**Fig 4:**
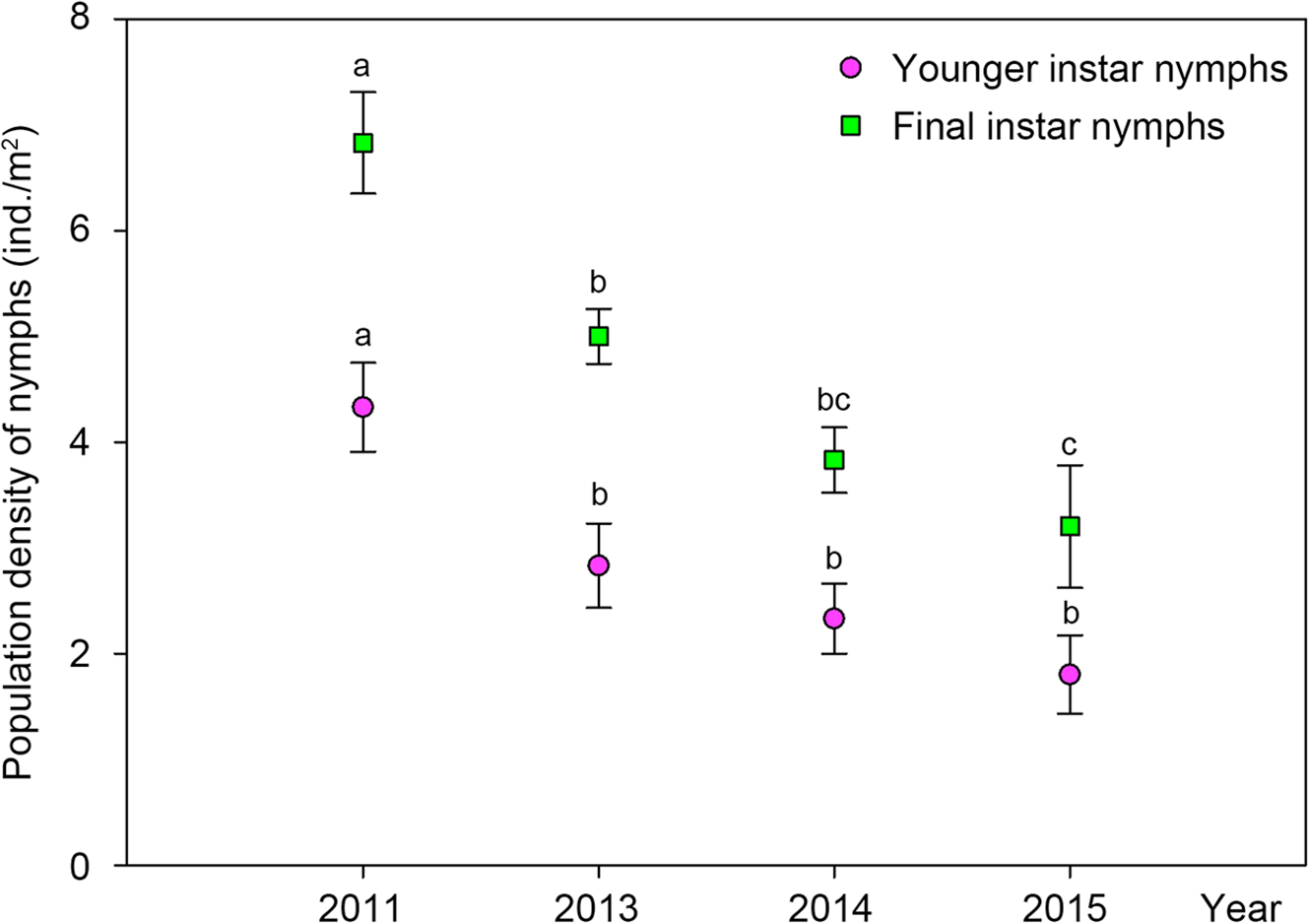
Population dynamics of nymphs of *C*. *atrata* feeding on *Populus tomentosa* in uncontaminated habitats. Different letters represent significant differences at the 0.05-level.

**Fig 5:**
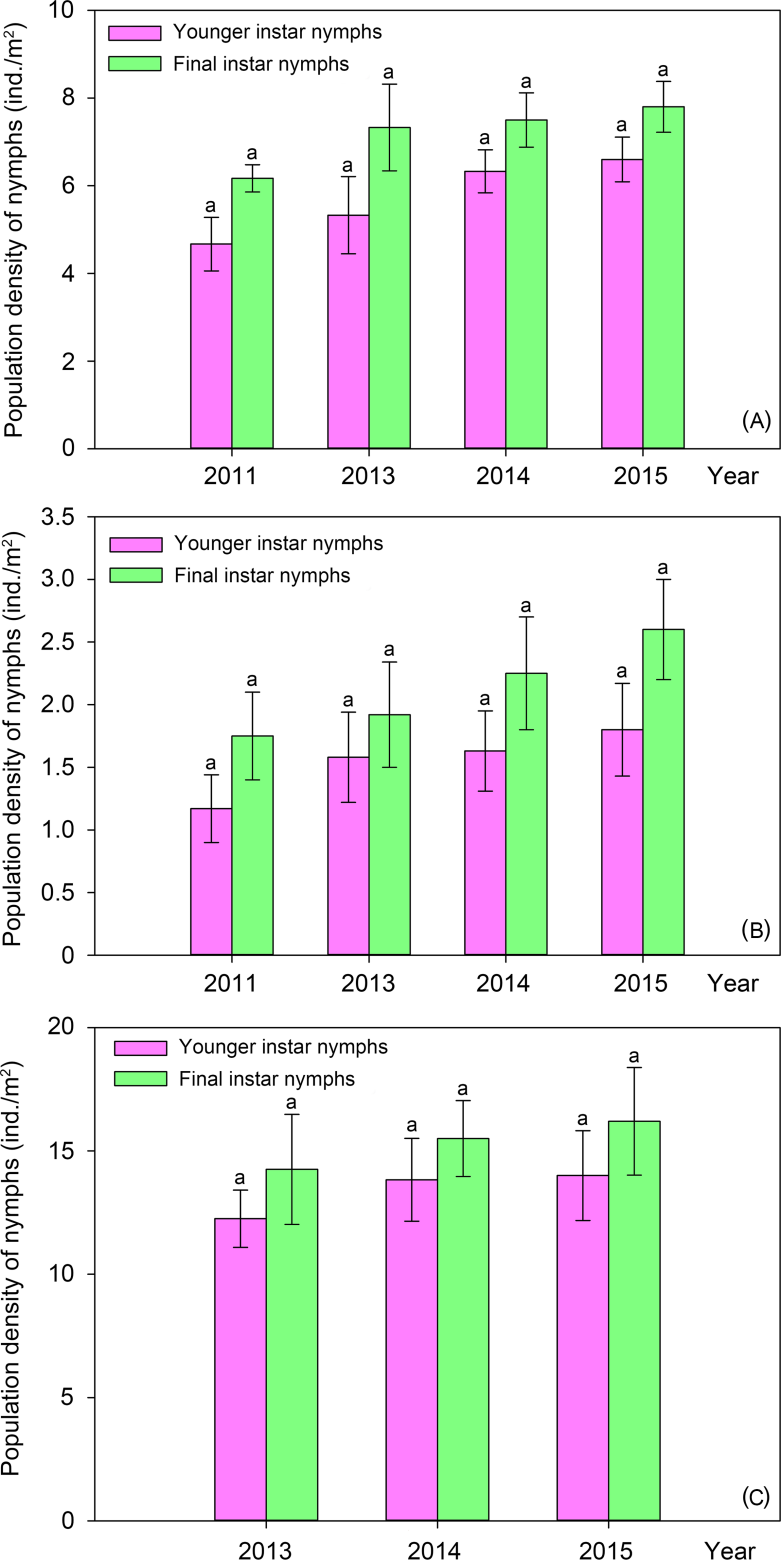
Population dynamics of nymphs of *M*. *mongolica* feeding on different host plants in uncontaminated habitats. (A) *Populus tomentosa*. (B) *Pinus tabuliformis*. (C) *Pyrus xerophila*. Different letters represent significant differences at the 0.05-level.

Under the host plant *Populus tomentosa* in the uncontaminated habitats, the population density of *C*. *atrata* nymphs distinctly decreased (Fig 4), with highly significant differences among the younger instar nymphs (ANOVA, F = 7.891, P = 0.001) and among the final instar nymphs (ANOVA, F = 14.900, P < 0.001) collected in different years. There was a distinct increasing trend of population of *M*. *mongolica*, i.e., populations of both the younger and final instar nymphs increased year by year (Fig 5A), although no significant differences were found neither among the younger instar nymphs (ANOVA, F = 1.834, P = 0.175) nor among the final instar nymphs (ANOVA, F = 1.109, P = 0.370).

**Fig 6:**
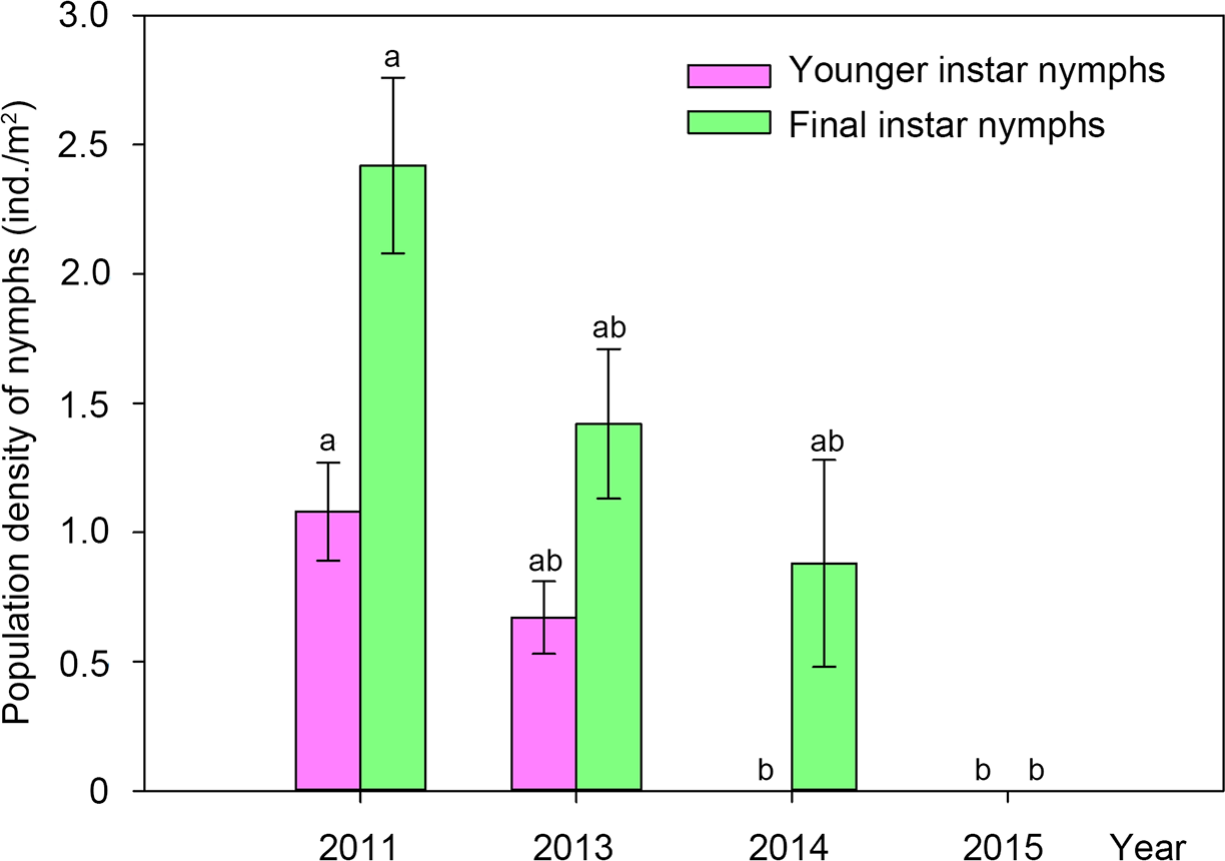
Population dynamics of nymphs of *P*. *kaempferi* feeding on *Pinus tabuliformis* in uncontaminated habitats. Different letters represent significant differences at the 0.05-level.

Under the host plant *Pinus tabuliformis* in the uncontaminated habitats, a slow trend of population increase was found in both the younger and final instar nymphs of *M*. *mongolica* (Fig 5B), although no significant differences were found neither among the younger instar nymphs (ANOVA, F = 0.619, P = 0.608) nor among the final instar nymphs (ANOVA, F = 0.634, P = 0.598). Conversely, in the same ecological niche, a distinct trend of population decline was found in the nymphs of *P*. *kaempferi* (Fig 6), with highly significant differences among the younger instar nymphs (Kruskal-Wallis test, H = 19.020, P < 0.001) and among the final instar nymphs (Kruskal-Wallis test, H = 15.319, P < 0.01). The younger instar nymphs of *P*. *kaempferi* could no longer be found since 2014, and the final instar nymphs of *P*. *kaempferi* could no longer be found either since 2015 (Fig 6).

Under the host plant *Pyrus xerophila* in the uncontaminated habitats, there was a distinct trend of population decline in both the younger instar nymphs and the final instar nymphs of *C*. *atrata*, and younger instar nymphs could no longer be found since 2014. Significant differences were found in the population density of the final instar nymphs (ANOVA, F = 4.465, P < 0.05) among 2013, 2014, and 2015. However, the population density of both the younger and final instar nymphs of *M*. *mongolica* increased year by year (Fig 5C), though no significant differences were found neither among the younger instar nymphs (ANOVA, F = 0.453, P = 0.643) nor among the final instar nymphs (ANOVA, F = 0.229, P = 0.798).

### Influence of C&D waste on fitness and morphology of cicada nymphs

According to morphological observations, three types of malformations were found from the malformed nymphs (mainly derived from the final instar nymphs of *M*. *mongolica*): 1) physically damaged individuals (still alive) (2, 3.2%) (Fig 2A), 2) fungus-infected individuals (40, 64.5%) (infected by entomogenous fungus *Elaphocordyceps* sp. (Fam. Ophiocordycipitaceae)) (Fig 2B–2E), and 3) bacterium-infected individuals (20, 32.3%) (Fig 2F).

**Fig 7:**
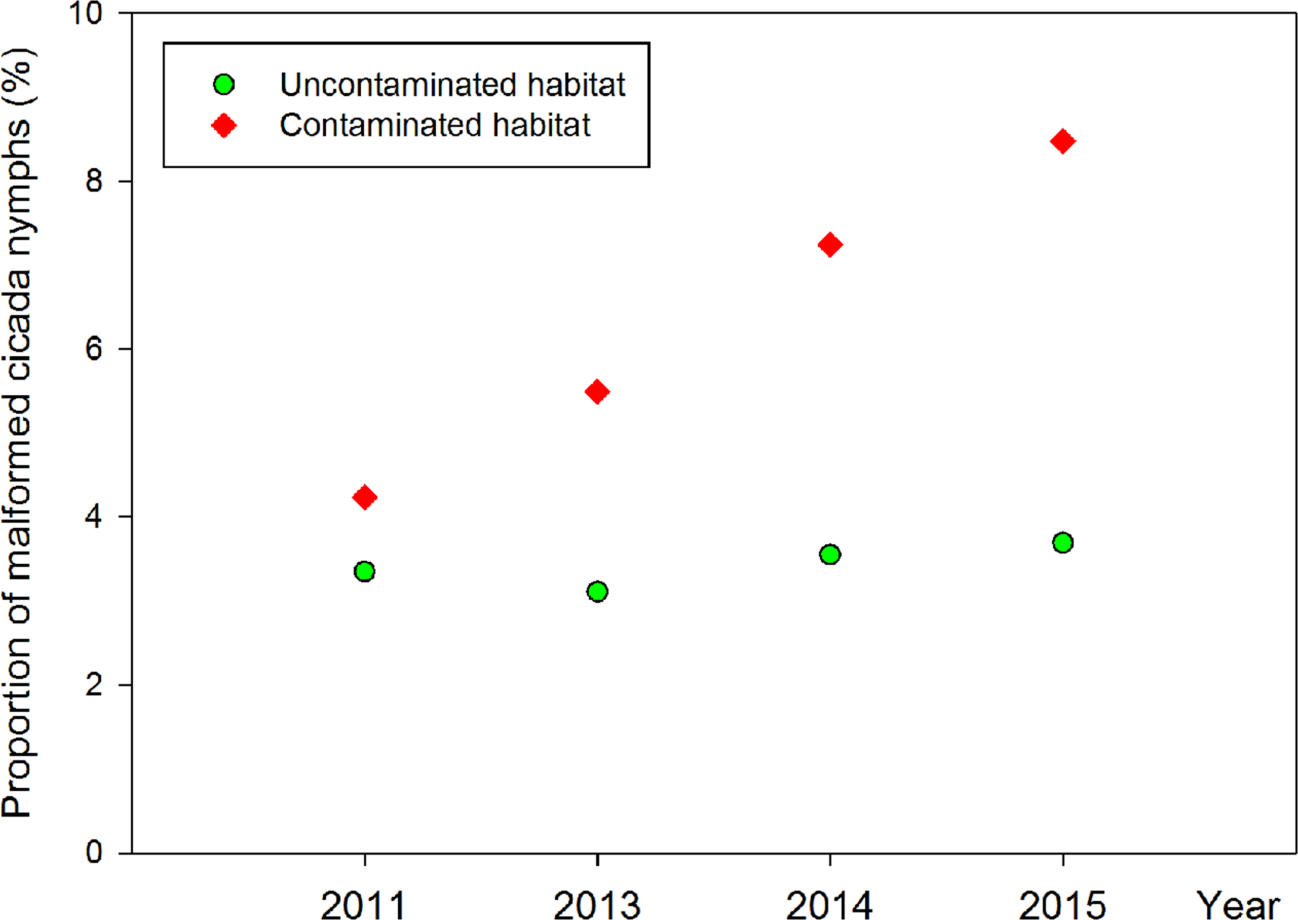
Proportion of malformed cicada nymphs feeding on *Populus tomentosa* in contaminated and uncontaminated habitats.

In the uncontaminated soil, the average proportion of malformed nymphs was about 3.4% (42/1236), with no significant differences among the four investigated years (2011: 7/209; 2013: 13/418; 2014: 12/338; 2015: 10/271) (Fisher’s exact test, P = 0.977) (Fig 7).

In the soil contaminated by C&D waste, 20 malformed nymphs were found among 337 individuals, with no significant differences among the four investigated years (2011: 5/118; 2013: 5/91; 2014: 5/69; 2015: 5/59) (Fisher’s exact test, P = 0.647). The proportion of malformed nymphs increased year by year (Table 2, Fig 7). Although the annual ratio of malformed individuals had no significant differences between the contaminated and uncontaminated habitats, the total percentage of malformed nymphs in the contaminated habitat is significantly higher than that in the uncontaminated habitat (Table 2). In addition, P-value of Fisher’s exact test for proportion of malformed nymphs between the contaminated habitats and the uncontaminated habitats in each year decreased year by year (2011: 0.762; 2013: 0.341; 2014: 0.183; 2015: 0.158). This indicates that the fitness of cicada nymphs in the contaminated habitats and the uncontaminated habitats existed differences, and that the differences become more significant as time goes by.

**Table 2:** Proportion of malformed cicada nymphs in different years.

Year	Contaminated habitats	Uncontaminated habitats	Fisher’s exact test P-value
2011	5/118	7/209	P = 0.762
2013	5/91	13/418	P = 0.341
2014	5/69	12/338	P = 0.183
2015	5/59	10/271	P = 0.158
Total	20/337	42/1236	P = 0.040

In addition to the malformations, morphological variations were also found in *M*. *mongolica* nymphs in the two different habitats. The abdomen of nymphs occurring in the uncontaminated soil was mainly white in color, while mainly greenish white in the contaminated soil.

## Discussion

The present study is the first to focus on the influence of C&D waste on cicada nymphs which are one of the most important and typical underground insects. The campus of Northwest A&F University was selected as the study site, because it represents a miniature of the rapid urbanization of this area. This campus has been severely polluted by a large amount of C&D waste, but little attentions have been paid to the threat of C&D waste to habitats yet.

We revealed a decline of population of cicada nymphs in the soil contaminated by C&D waste. Our results also indicate differences of fitness exist in the cicada nymphs between the contaminated habitats and the uncontaminated habitats, and the differences become more significant as time goes by. We infer that negative effects of cumulative consequences of C&D waste on the fitness and community structure of cicada nymphs strengthen year by year. The negative responses of cicada nymphs to the soil polluted by C&D waste have significant implications for the habitat destruction here. It indicates that cicada nymphs may be suitable bioindicators for underground-habitat-quality monitoring.

In our present study, *P*. *kaempferi* was not found from the soil under the host plants *Pyrus xerophila* and *Populus tomentosa*. On one hand, we revealed the distinct decline trend of populations of cicadas *C*. *atrata* and *P*. *kaempferi* living in the uncontaminated habitats. On the other hand, although no significant differences were found among the investigated years, yet we revealed a distinct increasing trend of population of *M*. *mongolica* in the uncontaminated habitats. This indicates that *M*. *mongolica* is possibly evolving into the most dominant species when it competes with the other two species in this area. The possible ecological succession of cicadas here may be closely related to the adaptability of related species to the adverse environments, but more researches are needed to establish whether there is a shift in the species composition.

Human mediation through different ecological disturbances could affect species interactions and foster displacement events [17]. For example, Holway hypothesized that “unnaturally” moist habitats in coastal California provide a competitive advantage to the invasive Argentine ant *Linepithema humile*: increased soil moisture through irrigation enabled *L*. *humile* to displace native ants that are adapted to more xeric habitats [18]. Reitz suggested that competitive displacement has the potential to be a widespread phenomenon, and these displacement events may increase in frequency [19]. Habitat fragmentation due to human activity has drastic effects on plant communities, mainly in terms of changes in the number of species and the structure and nutritional quality of plants [20], which has pronounced effects on herbivorous insects [21]. Herbivores with larger body size are often greatly affected by disturbances such as habitat fragmentation than the smaller ones, because they need more energy and resources in their life history. The body size of *M*. *mongolica* is smaller than that of *C*. *atrata* and *P*. *kaempferi* [14]. In our anatomical study, when living adults of the abovementioned three species were anesthetized by chilling at 4°C, adults of *M*. *mongolica* kept alive after several days, but individuals of the other two species all died. This indicates that *M*. *mongolica* has a higher tolerance and stronger viability in extreme environments to some degree. This is consistent with that the malformed nymphs found in our field investigation were mainly derived from the final-instar nymphs of *M*. *mongolica*, but much fewer malformed nymphs were found in the other two species whose population decreased year by year. They were mainly infected by an entomogenous fungus (*Elaphocordyceps* sp.), which is similar to the nymphs of the cicada *Karenia chama* Wei & Zhang [22]. One explanation for this phenomenon could be that the nymphs of *C*. *atrata* and *P*. *kaempferi* attacked by pathogenic microorganisms or affected by other adverse impacts in the contaminated soil could not survive in their early postnatal stages due to their weaker resistance, which eventually lead to the extinction of *P*. *kaempferi* in the soil under host plants *Pyrus xerophila* and *Populus tomentosa*. Besides that, the abdomen of 3^rd^- and 4^th^-instar nymphs of *M*. *mongolica* in habitats polluted by C&D waste were mostly greenish white, which is different with those living in the uncontaminated habitats. This implies that adaptive mechanisms may have evolved in *M*. *mongolica* against the severe trophic and living conditions, which is similar to that winged morph aphid could be produced in a nutrition poor environment [23]. Therefore, we conclude that *M*. *mongolica* is more competitive than other sympatric cicada species in our investigated area due to its stronger viability and flexibility.

Recently, there is growing evidence that belowground biodiversity has a major role in shaping aboveground biodiversity and sustaining ecosystem functioning [24]. In the present study, we show that the diversity of cicada nymphs is currently under the threat from anthropogenic pressures, e.g., soil pollution and habitat destruction caused by urbanization. Our results revealed that, besides the maintenance of their host plants, the maintenance of ecological health of habitats should be another crucial measure to conserve long-term subterranean insects like cicadas. It is urgent to build the amicable type of environmental development pattern and to construct a new ecological civilization in regions/countries with a rapid process of urbanization.

Our study also revealed that the negative responses of cicada nymphs to C&D waste have significant implications for habitat destruction, and that cicada nymphs may be suitable bioindicators for underground-habitat-quality monitoring. However, more research is needed to further address the association between the magnitude of pollution (e.g., degree, period and frequency of C&D waste contamination) and the fitness and population dynamics of cicada nymphs. Although our results suggest that *M*. *mongolica* nymphs might have developed some adaptive mechanisms in response to adverse conditions in habitats facing destruction by C&D waste, further study is required to establish the exact mechanisms to adapt to rapid changes in adverse environments (e.g., through phenotypic plasticity, range shifts, or by evolutionary adaptation), and whether they can do this fast enough to cope with the new environment.
